# COVID-19: Social Irresponsibility of Teenagers Towards the Second Wave in Spain

**DOI:** 10.2188/jea.JE20200360

**Published:** 2020-10-05

**Authors:** Maria Teresa Murillo-Llorente, Marcelino Perez-Bermejo

**Affiliations:** SONEV Research Group, School of Medicine and Health Sciences, Catholic University of Valencia, Valencia, Spain

As we already warned in the month of March, there was a rapid expansion of the Sars-CoV-2 coronavirus in Spain, mainly due to the inaction of the Government, allowing numerous mass demonstrations for demands or celebrations, despite knowing, as was shown later, the consequences of having allowed it.^1^

Once again, we write to denounce that a very high number of outbreaks is taking place in Spain, this time caused by social irresponsibility, mainly by the younger age groups. The vast majority of epidemic re-growths are associated with nightlife and occur in pubs, discos, and private parties in which the participants are young, and yet, after numerous interviews in the media, it is found not only that many young people continue to be aware of the severity of the pandemic, but affirm that they will continue to have fun without fear of the consequences that this may cause due to the spread of the virus.

This irresponsibility has meant that several countries in the European environment have canceled their flights to Spain or that some countries, such as the United Kingdom, have again imposed quarantine on travelers from Spain.^2^ In Spain, COVID-19 infections have exceeded 270,000 infections and fatalities already exceed 28,400, according to Johns Hopkins University.^3^

It is striking that, despite the vast information currently available, and the increasing scientific evidence of the consequences of the pandemic, young people continue to act in this way. A recent study states that nearly a third of young adults are medically vulnerable to severe COVID-19 disease, and that smoking is the strongest risk factor for young adults.^4^

The Spanish health authorities say they are concerned about the increase in infections among young people, attributing this fact to their way of socializing, in a “closer” way. This closeness, together with social irresponsibility, has caused a worrying variation in the data, going from 6% of infections in the first phase of the pandemic in young people between 15 and 29 years old to 19% today, having decreased the median age from 60 to 44 years, according to the data offered by the National Network of Epidemiological Surveillance,^5^ which also shows that the youngest are not as immune to the effects of the virus as previously thought.

This situation is beginning to occur also in other countries, such as Japan. A recent study shows that the majority of young adults who start outbreaks are asymptomatic, when they are not yet aware that they can infect other people, and they do so mainly in situations where they breathe hard and there is little social distance, such as singing in karaoke bars, screaming in pubs, or working out in gyms.^6^ The data found in this study suggests that young people can act as vectors of transmission of the virus to other age groups, which are the ones that suffer the greatest damage from COVID-19.

In this situation, there is an extremely urgent need to carry out awareness campaigns among adolescents, so that they are aware of the danger of acting so relaxed in the face of the security measures indicated by the health authorities, despite the fines and dissuasive actions of police and local authorities, who are desperately trying to avoid irresponsible mass concentrations of teenagers.
